# Lessons from Our Patients: Development of a Warm Autopsy Program

**DOI:** 10.1371/journal.pmed.0030234

**Published:** 2006-06-06

**Authors:** Kathleen Oare Lindell, Judith A Erlen, Naftali Kaminski

## Abstract

Kaminski and colleagues discuss the lessons they have learned in establishing a warm autopsy program to advance research on idiopathic pulmonary fibrosis.

Patients and their families can be our greatest teachers. Such was the case with the initiation of the Warm Autopsy Program at the Dorothy P. & Richard P. Simmons Center for Interstitial Lung Disease at the University of Pittsburgh. This brief essay describes events that led to our beginning the program, which we believe uniquely benefits researchers whose work focuses on the quest to further understand idiopathic pulmonary fibrosis (IPF). We outline the lessons that we have learned in the hope that our experience may be useful for other centers that are considering a warm autopsy program for patients and their families. A detailed description of the regulatory and ethical issues related to warm autopsy is beyond the scope of this essay and has been previously reviewed [ 1].

The idea for the Warm Autopsy Program was planted as one of our support group meetings was ending. One of the patients approached our clinical nurse specialist (KOL) privately, and said that he was “going to die soon” and wanted to donate his lungs to our program for research. He then said, “I don't want others who get this disease to suffer like me.” We had been holding a support group for patients with IPF for about two years, and this patient and his wife attended frequently. He was a retired firefighter who was unable to be seen in our clinical program because his insurance would not cover any medical expenses at our institution. His wife learned about our support group on the Internet and they actively participated. He had also been a trumpet player in a band and said that his participation in the band had decreased over the course of his illness. It was obvious that it was important to him to be able to donate his lungs to assist in finding a cure for this disease.

## Lesson One: Listen to the Patient

The support group leader (KOL) told him that our research team “would be happy to be able to use your lungs to further the research in this area, but would have to explore exactly how this could be done.” We had never had patients wanting to donate their lungs before and had not even discussed this possibility. KOL quickly approached the program director (NK), who said “this could be very important—there is little information about what happens in the lung when patients die from IPF.” NK then investigated how to proceed. Shortly thereafter, he shared that he had spoken to the head of the tissue bank in pathology, who reported that a similar program had been initiated with patients with prostate cancer [ 2].

Patients and their families can be our greatest teachers.

Further research showed that warm autopsy (also known as rapid autopsy) programs have been around for more than 25 years [ 1]. Most of these longstanding rapid autopsy programs focused on diseases such as Alzheimer disease and multiple sclerosis—where the organs are relatively inaccessible (they are not biopsied frequently from living patients), and therefore samples for research are rare [ 1]—but there were no programs for lung disease. Early work in organ transplantation paved the way for the ethical foundations of procuring organs [ 3]. The Uniform Anatomical Gift Act was adopted in the United States in the early 1970s to ensure that organ donation was an individual, free, and autonomous choice as the basis for donation from both living and cadaver sources [ 3]. Because international laws for consent for organ procurement, as well as attitudes toward organ donation, may differ in different countries and cultures [ 4], some aspect of these programs may be highly country- and culture-specific.

**Figure 1:**
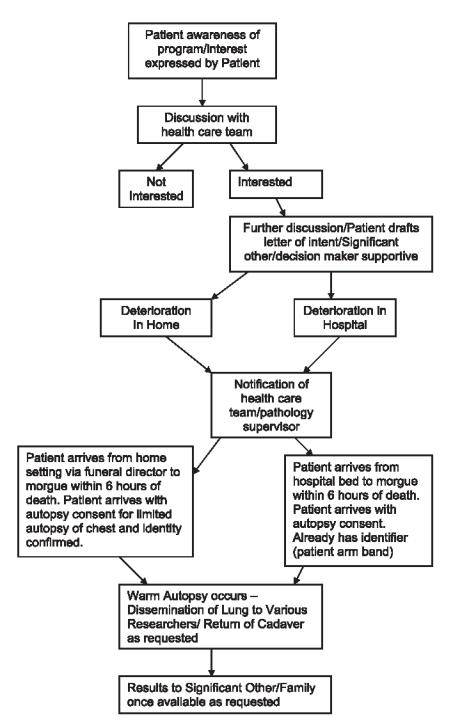
Warm Autopsy Process

We learned from the head of the tissue bank that for the lungs to be viable for research, they had to be received within six hours of death. This seemed reasonable until we considered our wide referral base. If death occurred in our hospital, the pathology technicians could obtain the lungs soon after the death. However, if the patient died at home or at an outside hospital, there were obvious constraints. We met with the supervisor of the tissue bank and worked out the details that would make the program work effectively ( Figure 1).

First, patients need to make their wishes known in writing and share these wishes with their significant other. At the time of their death, any written statement becomes null and void; as with organ donation, the patient's significant other needs to carry out the patient's wishes in order for the warm autopsy to take place. If the significant other decides not to pursue the patient's wishes, the warm autopsy does not occur.

The body also needs to arrive at the morgue with a consent form for the autopsy signed by the patient's significant other. This is potentially a logistical hurdle, depending on whether the body comes from within the hospital or from another setting. In a hospital setting, the consent forms for this limited autopsy are readily available. If the patient does not die at the hospital, however, the consent form may not be to hand. In this situation, the significant other should write a letter reiterating the patient's wishes, accompanied by the letter that the patient initially drafted; both documents need to accompany the body to the morgue. The warm autopsy cannot proceed at the morgue if there is no consent form with the body ( Figure 1).

We were also concerned about conducting research safely and properly within the institution's guidelines. Initially, we learned that a review by our institutional review board was not necessary, as these reviews apply only to research on living humans. However, there is a special committee, the Committee for Oversight of Research Involving the Dead, that reviews all research studies involving dead humans or tissue from dead humans. While not legally required, an approval by the committee certifies that all ethical concerns have been addressed [ 1].

## Lesson Two: Go to the People Who Have the Experience

Our research team was excited about the possibility of obtaining lungs from patients who died with IPF, because these donations would provide researchers with the opportunity to study fresh lung tissue. As such, there was the potential for new insights, but it was also imperative that as health-care providers we recognized and honored our moral obligations to the patients and their families to respect the wishes of the recently deceased.

The first opportunity for a warm autopsy took place in May 2003, when the team was at the American Thoracic Society International meeting in Seattle, Washington. The immediate reaction was to defer this autopsy and wait until the next opportunity. Fortunately, the patient's wife was persistent in making sure her husband's wishes were implemented. After several long-distance telephone calls and coordinated efforts of multiple members of the tissue bank, our staff, and a very supportive funeral director, the patient's body was transferred, complete with consent, from his home to the morgue in our hospital for the warm autopsy. Upon completion of the warm autopsy procedure, the chest wall was reattached and the body was returned to the funeral director to carry out the patient's burial wishes.

## Lesson Three: Family Members Are Often Your Best Allies

There are many benefits of the Warm Autopsy Program. The lungs can be studied by various researchers, including those whose work involves the airways, pulmonary arteries, lymph tissue, and individual cells. We have a team that looks at the gene networks in IPF by analyzing tissue samples from patients early on in their disease and pulmonary explants. Our ability to examine tissue from patients who have recently died may facilitate the ability to identify key events that led to the last deterioration. We share these findings with the family once they become available.

We believe this program strengthens the connection of the patients and the families to our Center. We also think that the program conveys the message that the team respects patients' wishes and allows them to contribute even in their last days. As an example, one patient died in the early evening, and as his son was going through his paperwork early the next morning, he read his father's request: “Please donate my lungs to the Simmons Center.” The son called us and relayed his father's request. Our research protocol required that lungs be harvested within six hours of death, but we wanted very much to respect the father's request. After a brief discussion, we identified some research uses that were not affected by the time that had passed. While rejection of these lungs would have been completely justified, we felt it was important to honor the patient's wishes and to try to allow the son to carry out his father's wishes.

## Lesson Four: Respect Your Patient's Last Wishes

As our support group has grown, the principles of honesty and confidentiality have been honored and are a mainstay of the meetings. The leader of a developing, growing group needs to be sensitive, flexible, and spontaneous in order to detect the participants' needs. Furthermore, the leader must promote group cohesion, offer structured information, ensure confidentiality, and strengthen honesty and spontaneity, as well as avoid subjective judgment of patients' concerns [ 5].

Patients with IPF want to know how their fellow group members are doing. We talk openly about advance care planning. Because patients often ask about individuals who are not present at a support group meeting, we request their permission to update others at the beginning of each session. We are honest about what has happened to their fellow group members. For example, at one meeting, the patients were told about the generous offers of a few of their peers to donate their lungs for the new Warm Autopsy Program. One of the patients exclaimed, “Who would have ever thought you would want these old scarred lungs!” A sense of levity was brought to a potentially awkward moment. In the discussion that followed, the patients talked about the practical and personal implications of being able to participate in this program.

The patients have created a network of E-mail addresses and telephone numbers and communicate regularly outside the group setting we provide. The patients are adept and independent in their activities, and are also a good source of support to each other; they often visit each other if hospitalized, and send cards and E-mails.

## Lesson Five: Allow Space for Patient Leadership

To date, we have had 12 patients participate in our Warm Autopsy Program, and we are currently analyzing the data obtained from this program. Preliminary analysis suggests that we are observing some fundamental mechanisms that could explain patient deterioration in IPF. Regardless of these results, the warm autopsy program has enriched our ties with our patients and strengthened our support group. It has provided us with an opportunity for our patients to exert their will in their last minutes and to feel that they may be making a major contribution to our understanding of IPF.

In a recent commentary in *Nature Medicine* [ 1], Pentz and colleagues provided ethical guidelines for warm autopsy programs. Interestingly, this commentary focused on the benefits to scientists and on the need to protect the patients and their families, but not on the role of patients and their families in this process. What we have learned from our patients is that participation in such a program can honor one of their last wishes. We also have learned that as health-care providers and scientists, we need to be alert to our patients' stories for innovative ideas and practices that address their needs. We hope that our experience will be useful to other groups, as they may encounter similar questions raised by their patients. As these programs develop, it is important that we partner with our patients and their families and allow them to take a leadership role in the development of such meaningful programs.

## Abbreviation

IPF: idiopathic pulmonary fibrosis
